# Twenty years of integrated disease surveillance and response in Sub-Saharan Africa: challenges and opportunities for effective management of infectious disease epidemics

**DOI:** 10.1186/s42522-021-00052-9

**Published:** 2021-11-09

**Authors:** Irene R. Mremi, Janeth George, Susan F. Rumisha, Calvin Sindato, Sharadhuli I. Kimera, Leonard E. G. Mboera

**Affiliations:** 1 Department of Veterinary Medicine and Public Health, Sokoine University of Agriculture, Morogoro, Tanzania; 2 SACIDS Foundation for One Health, Sokoine University of Agriculture, Morogoro, Tanzania; 3 National Institute for Medical Research, Dar es Salaam, Tanzania; 4 Malaria Atlas Project, Geospatial Health and Development, Telethon Kids Institute, West Perth, Australia; 5 National Institute for Medical Research, Tabora Research Centre, Tabora, Tanzania

**Keywords:** Disease surveillance, Data source, Performance, Big data, One Health, Sub-Saharan Africa

## Abstract

**Introduction:**

This systematic review aimed to analyse the performance of the Integrated Disease Surveillance and Response (IDSR) strategy in Sub-Saharan Africa (SSA) and how its implementation has embraced advancement in information technology, big data analytics techniques and wealth of data sources.

**Methods:**

HINARI, PubMed, and advanced Google Scholar databases were searched for eligible articles. The review followed the Preferred Reporting Items for Systematic Reviews and Meta-Analysis Protocols.

**Results:**

A total of 1,809 articles were identified and screened at two stages. Forty-five studies met the inclusion criteria, of which 35 were country-specific, seven covered the SSA region, and three covered 3–4 countries. Twenty-six studies assessed the IDSR core functions, 43 the support functions, while 24 addressed both functions. Most of the studies involved Tanzania (9), Ghana (6) and Uganda (5). The routine Health Management Information System (HMIS), which collects data from health care facilities, has remained the primary source of IDSR data. However, the system is characterised by inadequate data completeness, timeliness, quality, analysis and utilisation, and lack of integration of data from other sources. Under-use of advanced and big data analytical technologies in performing disease surveillance and relating multiple indicators minimises the optimisation of clinical and practice evidence-based decision-making.

**Conclusions:**

This review indicates that most countries in SSA rely mainly on traditional indicator-based disease surveillance utilising data from healthcare facilities with limited use of data from other sources. It is high time that SSA countries consider and adopt multi-sectoral, multi-disease and multi-indicator platforms that integrate other sources of health information to provide support to effective detection and prompt response to public health threats.

## Introduction

Despite scientific development to strengthen the health system to protect and promote human health, Sub-Saharan Africa (SSA) continues to be confronted by longstanding, emerging, and remerging infectious disease threats [1, 2]. The region vulnerability to infectious disease epidemics is driven by favourable climatic and ecological conditions for harbouring pathogens and their vectors in an environment with high human and animal interactions [3, 4]. Migration of wild animals and birds, frequent uncontrolled movements of people, commodities, animals and animal products across the national and international borders pose additional threats to the spread of infectious diseases [5]. Unfortunately, the region has a relatively low capacity for risk management of disease epidemics, mainly due to inadequate resources for early detection, identification, and prompt response [6, 7]. The failure in the early detection and response to epidemics in SSA is attributed to several factors, including deficiency in the development and implementation of surveillance and response systems against infectious disease outbreaks [8].

Before 1998, most countries in Africa implemented surveillance systems through vertical programmes of specific diseases of national and /or international priority. These included malaria, HIV/AIDS, tuberculosis and vaccine-preventable diseases. Epidemiological data were collected mainly at the health care facility level and in outreach health service settings [9, 10]. This situation led to fragmented and inefficient disease monitoring systems in many aspects, including resource allocation, flow and use of information and country capacity to detect and respond [9]. In response to an increased frequency of emerging and re-emerging diseases causing high morbidity and mortality in Africa, in 1998, the World Health Organisation (WHO) Regional Committee for Africa adopted a strategy called Integrated Disease Surveillance [9, 11]. The intent was to create and implement a comprehensive, integrated, action-oriented, district-focused public health surveillance for African countries [9]. In 2001 the strategy was renamed Integrated Disease Surveillance and Response (IDSR) to emphasise the critical linkage between surveillance and public health action and response [12].

IDSR functions are categorised into core and support functions. The core functions include identification of cases, investigation and confirmation, registration, case notification/reporting, data analysis and interpretation, response to the situation, communication and provision of two-way feedback, evaluation of the interventions, and preparation for emergency occurrences. The support functions include guidelines, laboratory capacity, supervision, training, resources and coordination at all health system levels [13]. The IDSR organisation structure allows surveillance information to flow from the low levels (community and facility) where data is generated through the district and national levels up to the World Health Organization. The IDSR implementation leverages the purpose and scope of the International Health Regulations 2005 [11].

During the past 20 years, the IDSR framework has been used in 94% (44/47) of the countries in the WHO African region to enhance capacity for surveillance for priority diseases, conditions, and events [14–16]. In most of these countries, the strategy has been implemented for about two decades, and the priority disease list required for reporting has been revised and increased [17]. Having a large number of diseases monitored by the public surveillance system creates implementation challenges. Low laboratory diagnostic capacity, low utilisation of the primary healthcare system and limited analytical skills and capacities in managing large and complex data result in unconfirmed and incomplete data and minimal utilisation of the data generated by the conventional system. Besides, the African continent has recently experienced major epidemics, including Ebola virus disease, dengue fever, cholera, yellow fever and coronavirus disease 2019, which spread faster and further due to high global connectivity, inadequate detection and risk management, and might easily be missed by the routine monitoring systems.

Over the years, the IDSR has relied heavily on the routine health management information system (HMIS) implemented at the facility and district levels of the health systems [16]. However, technology advancement and new communication platforms such as social and news media are growing in Africa, bringing more opportunities to incorporate digital data into surveillance information to complement passive facility-based surveillance. Since its adoption IDSR effectiveness and performance in SSA have been assessed, focusing on its functions. However, assessments on how the challenges and opportunities coming with IDSR evolved, how the technology expansion and the availability of other data sources relevant for surveillance have been embraced in monitoring, detecting and managing epidemics have not been documented with certainty [11, 14]. This systematic review aimed to analyse the performance of the IDSR strategy in Sub-Saharan Africa and how its implementation has embraced advancement in information technology, big data analytics techniques and wealth of data sources, as well as the One Health approach. The gaps, challenges and opportunities identified are used to propose appropriate strategies to improve surveillance in the region.

## Methods

### Search strategy and selection criteria

This review was guided by the following overarching question: Does IDSR generate information that drives early detection of and response to infectious disease outbreaks? Specific questions were: (i) Has IDSR improved health data quality and utilisation during its 20 years of implementation in SSA?; (ii) What are the challenges and opportunities for IDSR to improve early detection and prompt response to infectious diseases in SSA? The review followed the Preferred Reporting Items for Systematic Reviews and Meta-Analysis Protocols 2015 checklist [18]. Three databases, namely HINARI, PubMed, and advanced Google Scholar, were searched using Boolean operators. The search terms were Integrated Disease Surveillance and Response, Integrated Disease Surveillance, Health Management Information Systems, District Health Information System and Sub Saharan Africa or individual member country. The search was limited to studies published in the English language between January 1998 and December 2020. An additional search was conducted using the Google search engine on the World Wide Web and hand-searching from the reference list of the screened articles. Other sources were the World Health Organization (WHO), the United States Centres for Disease Control and Prevention, Africa Centre for Disease Control and Prevention and ministries websites of individual Sub-Saharan African countries.

The review involved two-stage screening, title/abstract screening and full-paper screening. The inclusion criteria were: the study must involve at least one of the SSA countries, clearly describe the evaluation of the IDSR system, focuses on at least one of the IDSR functions and/or systems attributes. The review excluded studies with abstracts without full text, not in English, reviews and newsletters. Two of the authors (IRM and LEGM) extracted eligible articles independently, and any disagreements between them on inclusion or exclusion were resolved by discussion and consensus. The linked descriptive search requests that were developed and search results from each database are presented in Table 1. Further exclusion of the article was performed during the data collection process after its full-text review. The extracted data related to the IDSR core and support functions’ performance, challenges associated with its implementation and improvement opportunities were summarised using the thematic analysis method.

**Table 1:** Search strategy and the number of articles included for screening

Database	Search strategy	Total results	No. Article included for screening	No. Article exclude
PubMed	(((Integrated disease surveillance and response) AND (Sub Saharan Africa)) OR (IDSR[Title/Abstract])) OR (Health management information system [Title/Abstract]) AND ((ffrft [Filter]) AND (journalarticle [Filter]) AND (fft [Filter]) AND (english [Filter]) AND (1998:2020[pdat]))	344	96	248
Hinari	((Integrated disease surveillance and response) OR (TitleCombined:(IDSR))) AND ((TitleCombined:(sub Saharan Africa)) OR (Health management information system))	1,052	302	750
Google scholar	((("Integrated disease surveillance and response" OR "IDSR" OR "Integrated disease surveillance") AND ("Health management information system" OR "district health information system")) AND "Sub Saharan Africa")	369	82	287
Other source	Integrated disease surveillance and response OR Integrated disease surveillance OR IDSR AND Sub Saharan Africa Health management information system OR district health information system	44	18	26
**Total**		**1,809**	**498**	**1,311**

## Results

### Literature selection

**Fig. 1:**
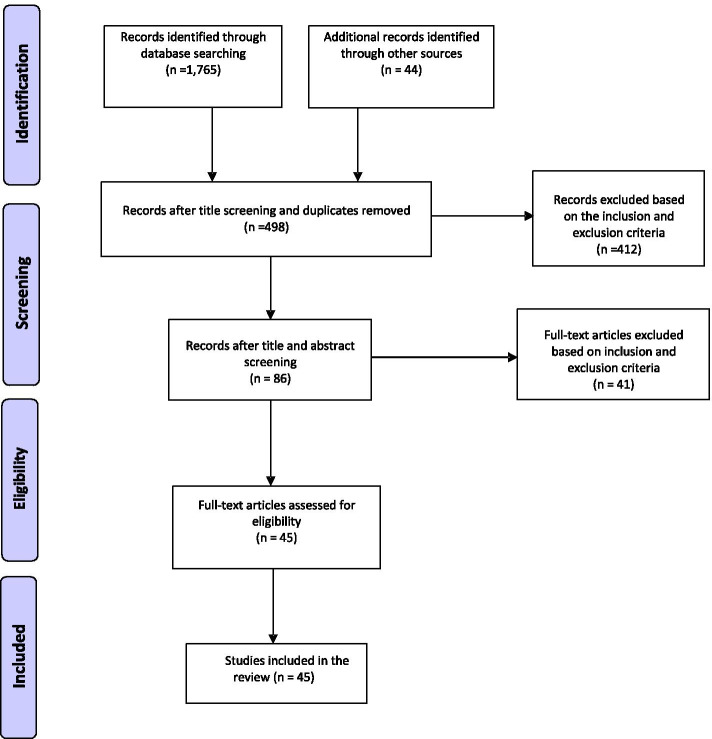
PRISMA flow diagram for article selection

A total of 1,809 articles were initially identified using the key search descriptors. A large number of articles (1,311) were irrelevant or duplicate and were excluded. The 498 remaining abstracts were screened further, and 412 were excluded based on the inclusion/exclusion criteria. Of the remaining 86, full-text articles were screened, and 45 studies met the inclusion criteria and hence, were selected for detailed reviews (Fig. 1). Of the 45 studies, 35 were country-specific, seven covered the SSA region, and three covered 3–4 countries. Of the 47 countries in Sub-Saharan Africa, country-specific studies were available for 20 (42.6%) countries. A total of 26 studies assessed the IDSR core functions, while 43 the support functions and 24 focused on either core or support functions. Twenty-four studies addressed both the core and support functions. Most of the studies involved Tanzania (9), followed by Ghana (6) and Uganda (5) (Table 2).

**Table 2:** Articles on IDSR core and support functions in Sub-Saharan Africa

Study country/ region	Core	Support	Reference
1. Africa	X	✔	[14]
2. Africa	✔	✔	[11]
3. Africa	✔	✔	[19]
4. Africa	X	✔	[20]
5. Africa	X	✔	[16]
6. Africa	X	✔	[21]
7. Africa	✔	✔	[8]
8. Côte d'Ivoire, Guinea-Bissau, Senegal, Mali	X	✔	[22]
9. Democratic Republic of the Congo	X	✔	[23]
10. Ethiopia	✔	✔	[24]
11. Ethiopia	✔	✔	[25]
12. Ghana	✔	✔	[26]
13. Ghana	✔	✔	[27]
14. Ghana	✔	X	[28]
15. Ghana	X	✔	[29]
16. Guinea	X	✔	[30]
17. Guinea	✔	✔	[31]
18. Kenya	X	✔	[32]
19. Kenya	X	✔	[33]
20. Liberia	✔	✔	[34]
21. Madagascar	X	✔	[35]
22. Malawi	X	✔	[36]
23. Nigeria	✔	✔	[37]
24. Nigeria	X	✔	[38]
25. Nigeria	✔	X	[39]
26. Nigeria	✔	✔	[40]
27. Rwanda	✔	✔	[41]
28. Sierra Leone	X	✔	[42]
29. Sudan	✔	✔	[43]
30. Tanzania	✔	✔	[44]
31. Tanzania	✔	✔	[45]
32. Tanzania	✔	✔	[46]
33. Tanzania	✔	✔	[47]
34. Tanzania	✔	✔	[48]
35. Tanzania	X	✔	[49]
36. Tanzania	✔	✔	[50]
37. Tanzania, Ghana	X	✔	[51]
38. Tanzania, Ghana, Uganda, Zimbabwe	X	✔	[15]
39. Uganda	X	✔	[52]
40. Uganda	✔	✔	[53]
41. Uganda	✔	✔	[54]
42. Uganda	✔	✔	[55]
43. Zambia	X	✔	[56]
44. Zambia	✔	✔	[57]
45. Zambia	✔	✔	[10]

Key: ✔=Article available; X= Article not available

Core functions included case detection, case confirmation; case registration; case reporting; data management; data analysis, outbreak preparedness, outbreak response, and feedback

Support functions included guidelines, laboratory capacity, supervision; training; resources (financial, human, material/equipment) and coordination

### Performance of IDSR strategy

The adoption and implementation of the IDSR strategy during the past 20 years have shown some improvements in several countries' disease surveillance activities. These include the integration of the surveillance functions of the categorical (or vertical) disease control programmes; implementation of standard surveillance, laboratory and response guidelines; improved timeliness and completeness of surveillance data, as well as increased national-level review and use of surveillance data for the response [14, 15]. However, most efforts to improve IDSR in SSA focused on the support functions rather than core functions. The successes of the desire for integration of the disease surveillance strategies in SSA have been documented in several countries, including Ghana, Ethiopia, Botswana, Kenya, Liberia, Sierra Leone, Uganda [56]. These include the efficient utilisation of the vertical programme surveillance mechanisms that provided functional infrastructure and trained personnel [56, 57].

### IDSR core functions

Improvements in IDSR system attributes such as completeness and timeliness of data reporting have been observed in Uganda, Malawi and Ghana [24, 35, 49, 51]. By the end of 2017, 68% of the countries in the WHO Africa Region had achieved the timeliness and completeness threshold of at least 80% of the reporting facilities. There was an improvement in timeliness of monthly and weekly reporting from 59 and 40% in 2012 to 93 and 68% in 2016, respectively [14]. During the same period of time, completeness of monthly and weekly reporting improved from 69 to 100% and 56 to 78%, respectively [14]. However, over the years, routine HMIS has remained the primary data source for IDSR in SSA. The routine HMIS in several SSA countries is characterised by persistent incompleteness and other data quality issues [58–60]. Studies in Ghana, Malawi, Mozambique, Nigeria and Tanzania have reported that case registration at a health care facility is also a challenge. There is a failure in comprehensively entering the appropriate patient information in the registers, and in some cases, diagnoses are either not recorded or wrongly recorded [60–64]. Moreover, high levels of mismatch between the register records and report forms and electronic District Health Information System-2 (DHIS-2) have been observed [60]. In Ethiopia and Liberia, IDSR data generated through HMIS were under-utilised due to poor data management and analysis skills [19, 22, 32]. A high level of mismatch between the HMIS registers' entries, tally sheets and the DHIS-2 database has also been reported in some countries in Africa [60, 65]. Thus, despite some progress in recent years, the core IDSR data source is still weak and inaccurately reflects what is generated from the primary healthcare facility levels [60].

Studies in Ghana, Tanzania, and Zambia have reported that several health facilities lack copies of the IDSR Technical Guidelines for Standard Case definitions; and that laboratories are ill-equipped to provide confirmation of any suspected priority notifiable infectious disease [10, 25, 60]. Lack of capacity for timely clinical screening, referral, diagnosis, notification, treatment and containment of suspected cases has been documented in Africa [66, 67]. Coordination of case definition reporting protocols across programmes was identified as a necessary step towards improving IDSR completeness and timely reporting in Uganda [52]. Moreover, since most primary level health care facilities lack diagnostic capabilities, the generated data rely on a syndromic approach, with low specificity [68]. Syndromic surveillance remains more useful at the community level for early detection and reporting of disease signals, which should be immediately verified and responded to by the primary health care facilities. Health care utilisation in many low-income countries is limited, and that only a proportion of people have access to conventional healthcare facilities. The utilisation frequency is higher among urban than rural populations. Several SSA countries have reported a frequency of between 40.0 and 87.3% of their population seeking care from conventional health care facilities [69–72].

### IDSR support functions

In terms of IDSR support functions, of the 47 countries in the WHO Africa Region, 94% were implementing the IDSR strategy, and 45 (85%) have initiated training at the sub-national level [14]. Thirty-three (70%) of the countries were using the electronic IDSR (eIDSR) system, and over two thirds (68%) had a feedback mechanism for sharing national surveillance data [14]. The introduction of the eIDSR using short message service for reporting weekly epidemiological data has proved to be a powerful tool that empowers health workers and addresses many of the barriers associated with paper-based reporting [38, 47, 52]. At the same time, the development of generic data analysis has guided enhanced data quality and management in Zimbabwe [73]. In terms of key performance indicators, there was a substantial increase in the number of countries that had adopted the IDSR guidelines and conducted training of healthcare workers at all levels [14].

## Discussion

### Challenges of IDSR

This review indicates that in most countries, data generated through the routine HMIS, which is the key source of IDSR, are rarely assessed for their quality, analysed and used to support decision-making [74]. Several studies in SSA have revealed weaknesses in case identification and recording at the primary healthcare facilities associated with several factors including limited skills among health workers due lack of training and refresher courses, patient-load versus human resource availability, low motivation and inadequate HMIS-related resources [15, 24, 44, 45, 49, 60]. The quality of the data remains a challenge, with incomplete and inconsistent data frequently being reported at different levels of the surveillance system. Moreover, HMIS data are considered to mainly reflect the population seeking care from health care facilities.

In Ethiopia, Liberia and Tanzania, assessments of the HMIS have identified some data quality issues and lack of use of the generated data [32, 43, 60, 75]. In a study in Ethiopia, though the surveillance system was found to be simple, useful, flexible, acceptable and representative, it lacked regular data analysis and feedback [22]. Moreover, studies in Kenya and Nigeria have indicated gaps between knowledge and practice of disease surveillance among health care workers [76, 77]. Incomplete data filing and inadequate organisation have been reported as an inbuilt shortcoming at all levels of IDSR in SSA [25, 26, 78]. Routine data analysis is still insufficient at facility and district levels in most countries, mainly due to the lack of clear guidelines for analysing data, shortage of skilled personnel, poor understanding of the use of surveillance data in planning, and inadequate infrastructure, including warehouses, computers, databases, data mining systems and analytical software [43, 44, 46, 51, 60].

A few countries (Burkina Faso, Ghana, Liberia, Uganda) have reported analysing and used routine HMIS data at sub-national levels [32, 74]. In both Liberia [32] and Tanzania [43], it was found that analysis and data use have not been given adequate attention. In addition to poor data management and analysis skills, some studies have reported under-utilisation of IDSR data at all levels due to poor data management and analysis skills [32, 42, 43, 60]. The culture of data analysis was lacking, and the relevance of surveillance data for decision making at sub-national levels was grossly underestimated. The use of paper-based reporting was likely to lead to severe limitations in the transmission of the data from the point of generation to a higher level mainly because of the inefficient report review and approval processes, manual routing of reports and running out of recording and reporting forms [25, 46]. Despite significant investment in early outbreak detection in SSA, there is very little evidence that even high HMIS data utilisation will influence early detection [79].

For the integrated system to be efficient, it requires strong coordination and communication, a clear organisation structure, adequate resources [80, 81], and reliable data sources. Integration may range from interconnectivity, which requires a simple transfer of files with basic applications, to complex convergent integration, which involves merging technology with processes, knowledge, and human performance. IDSR strategy strives for the concurrent integration route, but most countries have not achieved total integration. Implementation of the strategy is partially done [14, 35], and there is more focus on technical aspects than organisational and human resource issues hence impair the performance of the systems [49, 82]. Nevertheless, some countries such as Uganda have rectified those systemic challenges and reported improvement in the implementation [50].

## Opportunities for improving IDSR

### Health information systems

In SSA, several government ministries, agencies, and academic and research institutions are involved in managing different aspects of the health information systems. The ministries of health run the routine HMIS as the major source of information for decision making and planning. National Statistical Offices are responsible for most of the nation-wide household demographic and health surveys as well as population census [74]. Other key health-related information systems include civil registration, demographic surveillance sites and research outputs [83]. Demographic surveillance sites function in several countries, but the data generated are not integrated into the national health information system because of concerns about representativeness [74]. Besides, health research and academic institutions are increasingly generating evidence on human and animal health that could be used for disease surveillance purposes. However, most of the findings are mainly used for estimating national disease distribution rather than for planning national disease control programmes [84].

A warning of an impending epidemic can help relevant authorities and communities to prepare and take immediate actions to reduce morbidities and mortalities. Many of the epidemic diseases are highly sensitive to long-term changes in climate and short-term fluctuations in weather. Meteorological data are made available daily by the National Meteorological Agencies, yet they are rarely used to monitor the occurrence of diseases. Meteorological data can be combined with geospatially referenced data, population densities or road networks to generate estimates of environmental indicators relevant to infectious diseases [68]. However, such information is not available for planning, disease surveillance and outbreak management. It is recommended that the SSA governments consider establishing national platforms for infectious disease epidemics early warning systems and develop action plans for their operationalization, including resource mobilization and engagement with key stakeholders.

It is critical for a good and efficient surveillance system to incorporate other sources such as mortality data from demographic surveys, environmental data, vital statistics and civil registration, antimicrobial resistance, systematic surveys, meteorological data and research data. In most countries, despite an enormous amount of data generated by these systems, they run in parallel and independently, not well-coordinated, and sharing of information between them is minimal. Each of the existing systems operates its data collection and utilization framework. Moreover, much of the information is generated outside the health sectors – making it not readily available for disease surveillance purposes. It is a fact that the innovations, including the use of big data and artificial intelligence, could transform infectious disease surveillance and response and complement the existing traditional disease surveillance systems and improve detection and response to epidemics [68].

Laboratories play an important role in the prompt diagnosis of infectious diseases. The findings of this review have shown that IDSR is challenged by inadequate diagnostic capacities at all levels of the health system, especially in terms of staff levels, skill sets and infrastructure. It is critical, therefore, that countries support the efforts to strengthen laboratory capacities for the detection of a wide range of pathogens in relation to the IDSR priority diseases. Moreover, laboratory networking should be encouraged and should involve both national, regional and research reference laboratories. To address the gaps in knowledge, it is important to strengthen the laboratory management information systems (LIMS), recruit adequate staff who are well trained and motivated as well as the need for periodic support supervision of the surveillance activities. The plan by the African Centres for Disease Control and Prevention to establish and operationalize a Regional Integrated Surveillance and Laboratory Network is commended. This network is expected to coordinate and connect the continent’s analytical, surveillance, and emergency-response assets [85].

### Digital disease surveillance

An effective epidemic intelligence should contain both indicator-based and event-based surveillance. Globally, with the use of information technologies, an event-based surveillance approach is being promoted to complement the traditional “indicator-based” surveillance approach as part of the components of epidemic intelligence [86]. There have been growing interests in event-based internet bio-surveillance systems also referred to as digital disease surveillance (DDS) in recent years. DDS is the use of data generated outside the public health system for disease surveillance [86]. It involves the aggregation and analysis of data available on the internet, such as search engines, social media and mobile phones, and not directly associated with patient illnesses or medical encounters. It has been shown that digital approaches in surveillance improve the timeliness and depth of surveillance information in high-income countries [86, 87]. Recently, DDS has been used in responding to COVID-19 through case detection, contact tracing and isolation, and quarantine in several countries, including Taiwan, New Zealand and Thailand [88, 89]. In about 30 countries, algorithmic contact tracing through the use of a cell phone app or operating system has been deployed in response to the COVID-19 pandemic [89].

There is growing interest in using digital surveillance approaches to improve monitoring and control of infectious disease outbreaks [86]. However, such applications are scarce in Africa, and few studies have shown a direct connection between DDS and public health actions. So far, DDS has demonstrated its potential in early detection and response to Ebola and COVID-19 epidemics [90–95]. In a recent systematic review of the mobile-based infectious disease outbreak management systems (SORMAS) [93, 95, 96] was identified as having capacities to fully integrate data from case management, contact tracing, laboratory work and surveillance components. Currently, the Africa Centre’s for Disease Control and Prevention is implementing a pilot Programme in Ghana, Liberia, Madagascar, Nigeria, Sierra Leone and South Africa to develop digital surveillance indicators and online disease dashboards based on social media to inform infectious disease surveillance [97]. Moreover, there are ongoing efforts to create real-time data sharing platforms for disease surveillance using mobile technologies to allow centralized data management and use [96]. This is expected to strengthen real-time surveillance of infectious diseases in the continent, guide interventions, and build capacity in big data approaches for outbreak prediction, analysis and prevention.

With the proliferation of information technologies and increased ownership of mobile phones in SSA, there are large amounts of data on social media blogs, chatrooms, and local news reports that may provide governments and other stakeholders’ clues about disease outbreaks time and place daily. Such data are essential raw materials for DDS. Advancements in information technology and information sharing give rise to infodemiology – defined as the science of distribution and determinants of information in an electronic medium, specifically the internet [90]. To date, Program for Monitoring Emerging Diseases (ProMED-mail) [98] and HealthMap [99] are among the several leading efforts in digital surveillance. The World Health Organization routinely uses HealthMap, ProMED and similar systems to monitor infectious disease outbreaks and inform public health officials and the general public [100]. The key advantages of DDS include speed and volume, which may increasingly help health officials to spot outbreaks quickly and cheaply [96].

### Community event-based surveillance

Community-based surveillance (CBS) is defined as the systematic detection and reporting of events of public health significance within a community by community members [101]. Community engagement has long been an essential part of both human and animal health [102–105]. CBS has played a significant role in smallpox, guinea worm and polio eradication programmes [103]. Recently, CBS was reported as an important component in response to the West African Ebola virus disease outbreak of 2014–2016, where community health workers and volunteers worked together in early detection and timely reporting to the health system [106]. With CBS, public engagement is being transformed through participatory surveillance systems that enable the community to directly report disease events via information technology and communication tools [107]. Several CBS systems have been described and demonstrated their accuracy and sensitivity, their ability to provide more timely disease activity measures, and their usefulness in identifying risk groups, assessing the burden of illness and informing disease transmission models [108, 109]. CBS can provide early warning for emerging events by engaging the communities to detect potential public health events and connecting individuals to health services [3, 110, 111]. In a study in Ivory Coast, following the implementation of CBS, 5 to eightfold increases in reporting of suspected measles and yellow fever clusters have been reported [110]. These findings suggest that CBS can be used to strengthen the detection and reporting capabilities for several suspect priority diseases and events.

The WHO Technical Guidelines on IDSR [13, 17] highlight the need for CBS. This is because most of the health problems and events happen at the community level, thereby placing the community as the primary sensor of the disease signals. Thus, putting a surveillance mechanism to obtain information at the community level is an added advantage to capture diseases and public health events at their early stages to allow effective preparedness and response, thereby managing disease outbreaks at the source. Despite the relevance of the inclusion of community information in surveillance, by the end of 2017, only 32 (68%) of the 44 countries in the WHO Africa region had commenced CBS, and 35 (74%) had event-based surveillance [14]. However, there is only one report from Sierra Leone that indicates data collected from the two approaches are integrated into the national IDSR system [110]. In some countries, the CBS Programme are still operating as pilot or research projects [112, 113], and most cover a limited geographical area and are mainly for specific disease programmes in rural settings [110].

### One health surveillance

As part of an effective global response to diseases transmitted between animals and humans [114], there have been calls for integrating surveillance of zoonotic diseases in human and animal populations. The driving force is that about three-quarters of humans’ emerging infectious diseases have animal origin [115]. One health (OH) concept promotes the multi-sectoral collaboration between human, animal, and environmental health disciplines and sectors in addressing complex health issues [114]. Several African countries have carried out their prioritisation exercises on the zoonoses. Among the diseases that were ranked high include anthrax, brucellosis, viral haemorrhagic fevers, zoonotic avian influenza, human African trypanosomiasis, rabies and plague [116–120]. With this approach, OH surveillance is strongly encouraged at all levels to efficiently manage and coordinate health events involving humans, animals and their environment [16]. However, there are issues that need to be considered and addressed in the adoption of OH surveillance. These include the need to define the characteristics of OH surveillance and identify the appropriate mechanisms for inter-sectoral and multi-disciplinary collaboration [81, 116].

In 2019, the Tripartite organisations – the Food and Agriculture Organization of the United Nations, the World Organisation for Animal Health, and the World Health Organization – developed the Tripartite Zoonoses Guide (TZG). The aim is to help the countries develop a capacity to address zoonoses in a coordinated manner, linking to existing international policies and frameworks and supporting efforts for global health security. The TZG includes three operational tools to support national authorities: (i) the Multi-sectoral Coordination Mechanism, (ii) the Joint Risk Assessment, and (iii) the Surveillance and Information Sharing operation tools [121].

### Towards multi-sectoral and multi-indicator surveillance

**Fig. 2:**
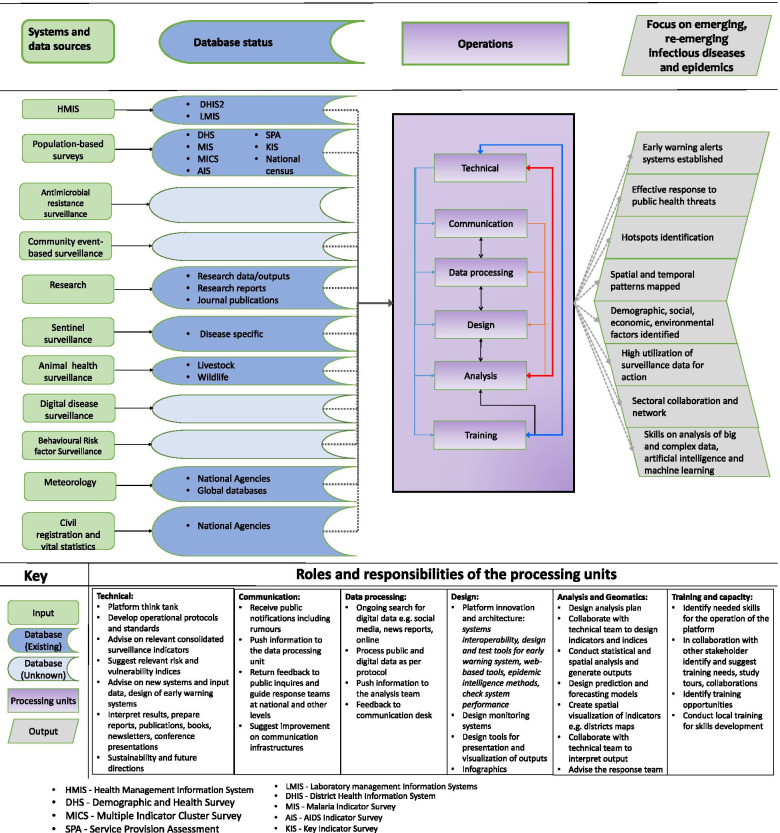
National Platform for a Multi-Sectoral, Multi-Disease and Multi-Indicator (3Ms) Epidemic Intelligence System

The emerging and re-emerging infectious diseases in Africa underline the urgent need to integrate public health surveillance systems [122]. As infectious disease threats increase in SSA, effective ways of predicting outbreaks and planning for outbreak responses become increasingly important. However, an epidemic intelligence that encompasses early warning functions for infectious diseases of humans and animals in SSA is almost non-existent. Therefore, we propose the development and adoption of a national platform for public health surveillance that is a multi-sectoral, multi-disease and multi-indicator epidemic intelligence system (Fig. 2). The system is envisaged to consolidate information from existing surveillance systems to define composite surveillance indicators with intelligence to trigger and guide unified responses to public health threats across sectors and diseases that share common risks. In One Health perspective, such a system may reduce the hurdle of monitoring enormous sector-specific and single-disease indicators, strengthen multi-sectoral collaboration, improve data quality and ultimately IDSR performance. For its operationalisation, a multi-sectoral coordination mechanism with representatives from the sectoral ministries should be established with timely-defined rotational leadership between the sectors responsible for human, animal and environmental health.

The importance of using both formal and informal data sources for timely and accurate infectious disease outbreak surveillance has been emphasized [86]. Evidence-based outbreak preparedness provides ground to streamline and concentrate efforts towards diseases that have been documented to circulate. Among other things, outbreak preparedness entails predicting possible epidemics with regards to the possible location of involvement, the risk and vulnerability of the population, the extent of the outbreak, its spread and socioeconomic consequences. Therefore, for any effective outbreak preparedness plan, information on prior risks is crucial in setting robust outbreak management and response plans. Research findings for decades have displayed mapping of exposure patterns and the burden of infectious diseases that can cause outbreaks in the community [5].

Modern technologies such as artificial intelligence and machine learning are widely applied in analysing a significant volume of data to assess the status and forecast future dynamics of diseases [123, 124]. A number of prediction models have been developed to provide event prediction, special ecological niche, diagnostic or clinical, spread or response information. The prediction models are valuable for disease prevention and saving disability-adjusted life years [125]. They also save valuable financial resources due to the high costs and resource utilisation associated with traditional surveillance systems. These emerging technologies are likely to become powerful means of facilitating the collection of more accurate and timely information, leading to information-based evidence. The techniques are expected to allow decision-makers to identify areas where the model predicts a particular risk category with certainty to effectively target limited resources to those areas most at risk for a given season.

## Conclusion

This review indicates that most countries in SSA rely mainly on traditional indicator-based disease surveillance utilising data from healthcare facilities with limited use of data from other sources. However, the traditional indicator-based disease surveillance approaches face several challenges, including data quality and inefficient early warning systems, because they are less sensitive than event-based surveillance approaches. They most often miss information from populations who do not access health care or do so through informal channels, thus unable to detect new, potentially high-impact disease outbreaks. Moreover, there is a dearth of information on IDSR data quality, analyses that utilise advanced methodologies and use in the detection and response of infectious disease outbreaks in the region. Over the years, data-use and data-process have not been given adequate attention. This analysis indicates that future efforts to address disease surveillance systems should consider data quality, multi-source data analysis and triangulation, data use and data integration. Capacity building for health workers at the national and sub-national levels in data management is critical.

This review highlights the untapped opportunities for integrating community-based, digital surveillance through a one health approach that could improve public health surveillance in SSA. It is high time that the region explores and adopts the integration of several surveillance programmes into hybrid systems that combine traditional surveillance data with data from the public health laboratories, community, research settings, search queries, social media posts, and crowdsourcing. Improved performance requires the merging of current gains, strong collaboration from all stakeholders, supervision and regular evaluation of the surveillance system to identify and address challenges as they emerge. The introduction of innovative ways to further strengthen the surveillance and response system in SSA countries is critical to enhancing early detection and reporting of suspected cases of priority diseases, conditions and events.

To address the challenges of the IDSR system, there is a need to develop an electronic platform that will combine data from multiple relevant databases such as HMIS, research programmes, laboratory management information systems (LMIS), population-based surveys, digital disease surveillance, sentinel surveillance, OH surveillance and community-based surveillance initiatives to allow their interoperability. The aim is to make optimal use of community, facility and research-based epidemiological information in preparing the community to act before a health emergency happens, as well as to provide high-quality evidence to guide policy development and resource allocation at the national level. With this platform, a continuing analysis and review of scientific publications, social media, routine health data and demographic statistics can be established to feed different decision-making units. Composite and multi-sourced indicators that comprise information from various sources can be generated, analysed and monitored. The goal is to make data readily available and help speed up dissecting the information and putting programmes in place to detect and promptly respond to epidemics. The platform will foster improved utilisation of surveillance data for action and avoid delays in response to emergencies by linking health indicators with other information such as climate data that can add value to inform health risks accurately. A multi-sectoral approach should be used to pursue a common strategic goal of developing a workforce that can support public health surveillance and response.

## Data Availability

All data relevant to the study are included in the article. No data are stored in a repository. No unpublished data are available following this review.

## Abbreviations

CBS: Community-based Surveillance
CDC: Centres for Disease Control and Prevention
DDS: Digital disease surveillance
DHIS: District Health Information System
HIS: Health Information Systems
HMIS: Health Management Information System
ICT: Information and Communication Technology
IDS: Integrated Disease Surveillance
IDSR: Integrated Disease Surveillance and Response
OH: One Health
PRISMA-P: Preferred Reporting Items for Systematic Reviews and Meta-Analysis Protocols
SMS: Short Message Service
SSA: Sub-Saharan Africa
USSD: Unstructured Supplementary Service Data
WHO: World Health Organization
